# Heterologous protein production in filamentous fungi

**DOI:** 10.1007/s00253-023-12660-8

**Published:** 2023-07-05

**Authors:** Dujuan Liu, Sandra Garrigues, Ronald P. de Vries

**Affiliations:** 1 Fungal Physiology, Westerdijk Fungal Biodiversity Institute & Fungal Molecular Physiology, Utrecht University, Uppsalalaan 8, 3584 CT Utrecht, The Netherlands; 2 Present Address: Department of Food Biotechnology, Instituto de Agroquímica Y Tecnología de Alimentos (IATA), Consejo Superior de Investigaciones Científicas (CSIC), Paterna, Valencia Spain

**Keywords:** Filamentous fungi, Heterologous protein production, Fungal cell factories, Gene regulation

## Abstract

**Abstract:**

Filamentous fungi are able to produce a wide range of valuable proteins and enzymes for many industrial applications. Recent advances in fungal genomics and experimental technologies are rapidly changing the approaches for the development and use of filamentous fungi as hosts for the production of both homologous and heterologous proteins. In this review, we highlight the benefits and challenges of using filamentous fungi for the production of heterologous proteins. We review various techniques commonly employed to improve the heterologous protein production in filamentous fungi, such as strong and inducible promoters, codon optimization, more efficient signal peptides for secretion, carrier proteins, engineering of glycosylation sites, regulation of the unfolded protein response and endoplasmic reticulum associated protein degradation, optimization of the intracellular transport process, regulation of unconventional protein secretion, and construction of protease-deficient strains.

**Key points:**

• *This review updates the knowledge on heterologous protein production in filamentous fungi.*

• *Several fungal cell factories and potential candidates are discussed.*

• *Insights into improving heterologous gene expression are given.*

## Introduction

Filamentous fungi have been used to produce a wide range of valuable compounds for centuries. With the advent of biotechnology and molecular (synthetic) biology, they have been exploited as hosts for the production of primary and secondary metabolites, and homologous and heterologous proteins which are worth several billion dollars per year (Cairns et al. 2018). Filamentous fungi have several advantages over other microorganisms in terms of homologous protein secretion. For instance, they have a natural ability to secrete a variety of proteins in large quantities, efficient folding, post-translational modifications, inexpensive cultivation, and easy induction (Madhavan et al. 2022). Furthermore, industrial fermentation systems are well established for several species. However, heterologous protein production in filamentous fungi is far from optimal and there is still scope for further improvement (Meyer et al. 2016). At present, compared to homologous proteins, the level of heterologous protein production is significantly lower (Li et al. 2022). In case of the industrial workhorse *Aspergillus* *niger*, for example, the production level of a homologous protein has been reported to be up to 400 times higher than a heterologous protein (Li et al. 2022)*.*

**Fig. 1:**
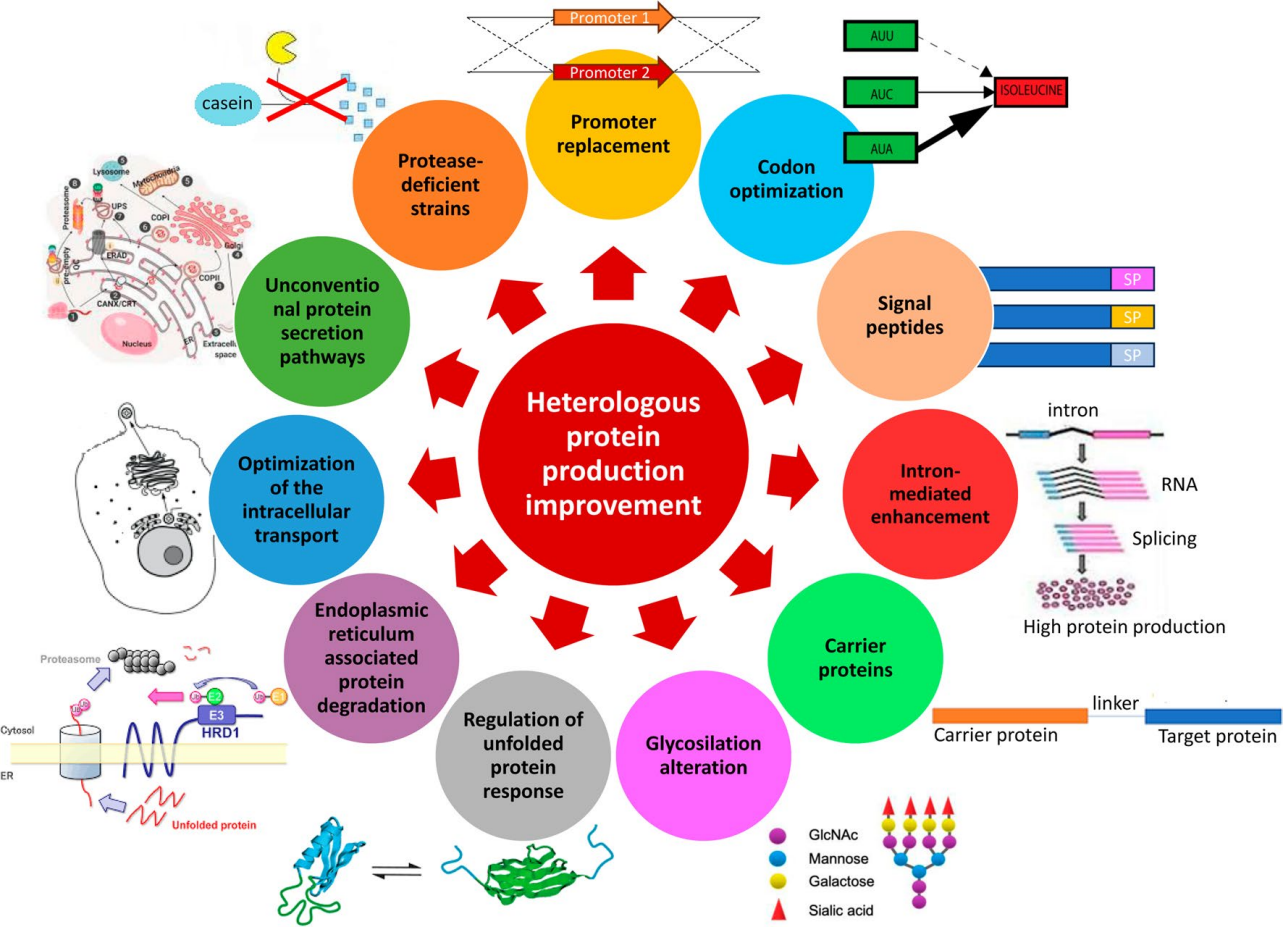
Approaches used for improving the production of heterologous proteins in filamentous fungi

Mainly species of the genera *Aspergillus*, *Trichoderma*, *Penicillium*, and *Myceliophthora*, and to a lesser extent *Fusarium*, *Rhizopus*, or *Mucor*, are being used for large-scale protein production (Meyer et al. 2020). Furthermore, species such as *A.* *niger*, *Aspergillus oryzae*, *Penicillium chrysogenum*, or *Trichoderma reesei* have a Generally Recognized As Safe (GRAS) status, facilitating their industrial applications. In fact, according to the list of enzymes compiled by the Association of Manufactures and Formulators of Enzymes Products updated in May 2015, the main fungal hosts used for the production of industrial enzymes are *Aspergillus* and *Trichoderma* species, followed by *Penicillium* (AMFEP, https://amfep.org/_library/_files/Amfep_List_of_Enzymes_update_May_2015.pdf). Many enzymes derived from these species have been applied in several industrial areas such as food and feed, biofuels and biochemicals, pharmaceutics, pulp and paper, detergents, textile, waste management, and/or agricultural industries (Kalra et al. 2020). In this review, we aim to highlight recent advances in fungal cell factory research and development, with a particular focus on the approaches used for improving the production of heterologous proteins in filamentous fungi, which are summarized in Fig. 1.

## Main fungi used as protein cell factories

### *Aspergillus*

Species in the genus *Aspergillu*s are relevant to diverse fields such as biomedicine, bioenergy, health, and biotechnology. This is a large and diverse genus comprising ~ 400 recognized species including the industrially important *A.* *niger*, *A. oryzae*, *Aspergillus* *sojae*, *Aspergillus tubingensis*, *Aspergillus terreus*, and *Aspergillus unguis*, and some pathogenic species such as *Aspergillus fumigatus* and *Aspergillus* *flavus*, which are harmful to animals and/or plants (Romero et al. 2021)*. Aspergillus* species are effective decomposers of organic substrates, as they are generally very efficient producers of extracellular enzymes. Moreover, *Aspergillus* species have become very suitable fungal cell factories at the industrial level due to their good ability to produce and secrete native and heterologous proteins, organic acids, and secondary metabolites (Ntana et al. 2020). In fact, among the commercial enzymes used in the food industry that are produced in filamentous fungi, more than 50% are produced in aspergilli (https://amfep.org/_library/_files/Amfep_List_of_Enzymes_update_May_2015.pdf).

*A. niger* is widely used as an industrial workhorse for the production of organic acids (particularly citric acid), proteins, and for basic genetic research (Yang et al. 2017; Lübeck and Lübeck 2022). In the last decades, much progress has been made for protein production using *A.* *niger*, with increasing titers of secreted proteins. Additionally, *A.* *niger* and other aspergilli have been harnessed for biosynthesis of diverse enzyme cocktails (Cairns et al. 2018). As examples, phytases, which are used to improve the nutritional content of animal feed, were first marketed in 1991 (Jatuwong et al. 2020). The biotechnological market of phytases is estimated to be around €150 million per year, with *A.* *niger* being one of the most commonly used microorganisms for their production (Haefner et al. 2005). Moreover, the production level of recombinant *Myceliophthora thermophila* trehalase and *Penicillium citrinum* nuclease P1 achieved 1698.83 U/ml (Dong et al. 2020) and 77.6 U/ml (Chen et al. 2019), respectively, in *A.* *niger*, and the yield of human erythropoietin was 73.9 mg/l, which is a 41-fold improvement over the original yield (Rojas-Sánchez et al. 2020).

*A. oryzae* is together with *A.* *niger* one of the preferred hosts for the production of heterologous proteins due to its excellent protein secretion capabilities. Its use is also considered safe as it has been used in fermented cuisine (miso, soy sauce, or douchi) for more than 10 centuries (Jin et al. 2021). One of the advantages of *A. oryzae* is that it secretes large amounts of enzymes but produces very few secondary metabolites under typical cultural conditions (Meng et al. 2022). The β-glucosidase (BGL) from the lower termite *Neotermes koshunensis*, a well-known cellulose decomposer, was successfully produced by *A. oryzae* with a 48-fold increase (Uchima et al. 2011). Moreover, the bovine chymosin (CHY), human lysozyme (HLY), and recombinant antibodies (such as adalimumab) were also produced by *A. oryzae* to decrease their production costs (Daba et al. 2021).

*Aspergillus vadensis* is a new species of black aspergilli which does not acidify the growth medium and produces very low levels of extracellular protease activity, both of which contribute to an increased stability of the protein of choice in the culture broth of this fungal species (de Vries et al. 2005). Additionally, it has a very high transformation frequency, which is convenient for the high-throughput screening of transformations (De Vries et al. 2004). These characteristics make *A. vadensis* a very promising candidate as a host for protein production. For example, the feruloyl esterase B from *A.* *niger* was synthesized in *A. vadensis* at a 12-fold higher level than the *A. niger* overproducing strain (Alberto et al. 2009). In addition, 11 different promoters for heterologous protein-encoding gene expression in *A. vadensis* have been tested and their efficacy has been addressed (Culleton et al. 2014).

There are also other *Aspergillus* species that have been developed into heterologous protein production platforms. For instance, the suitability of *A. unguis* as a host for heterologous protein production through the expression of the pharmaceutically important human interferon gene has been demonstrated (Madhavan et al. 2017). A gene coding for an *α*-galactosidase enzyme from *A.* *fumigatus* has been expressed in *A. sojae*, resulting in approximately 3-fold higher production (Gürkök et al. 2010). The extracellular endo-β-1,4-mannanase gene from *A. fumigatus* was also successfully expressed in *A. sojae* with a 12-fold increase (Duruksu et al. 2009).

On the other hand, *Aspergillus nidulans*, which is not a species commonly used in industry but a model organism for eukaryotic research, has been used for the heterologous production of Carbohydrate Active enZymes (CAZymes) with promising results (Kumar 2020). Using mass spectrometry-based proteomic approaches, Zubieta et al. demonstrated that *A. nidulans* is indeed suitable for high expression and production of heterologous proteins by analyzing its intracellular proteome (Zubieta et al. 2018). Endoxylanase- and arabinofuranosidase-encoding genes from *Penicillium funiculosum* and *A.* *niger*, respectively, were introduced into *A. nidulans*, yielding 301.2 U/mg and 115.55 U/mg, respectively (Gonçalves et al. 2012).

### *Trichoderma*

*Trichoderma* spp. are good hosts for heterologous gene expression because of their saprotrophic and mycoparasitic lifestyles, which allow them to grow on a diverse range of nutrients (Tomico-Cuenca et al. 2021). The majority of the cellulolytic enzymes utilized today in the biomass to biofuels or bioproducts industry are produced by *T. reesei* (*Hypocrea jecorina*) (Singh et al. 2015). Production of heterologous proteins in *T.* *reesei* has been carried out for decades, dating back to 1989, when calf chymosin was produced in this species (Harkki et al. 1989). With the genetic engineering of the strains and optimized culture conditions, up to 1 g/l of BGL from *A. terreus* was produced by *T.* *reesei* in a shake-flask culture (Wei et al. 2013). Also, 4.5 g/l human interferon alpha-2b could be produced by *T. reesei* in a bioreactor (Landowski et al. 2016). The extremely efficient protein synthesis machinery of *T*. reesei allows homologous protein yields higher than 100 g/l (Rantasalo et al. 2019). However, the yields for heterologous protein production remain modest (Jørgensen et al. 2014). Methods for improving heterologous protein production in this fungus are thus extremely desirable.

### *Penicillium*

*Penicillium* species are predominantly saprobic in nature, and several species have been used in commercial food production (e.g., dairy products) and the manufacturing of the antibiotic penicillin, among others (Toghueo and Boyom 2020).

*P. chrysogenum* (*Penicillium rubens*) has significant industrial importance and is a well-studied model for protein secretion with GRAS status. The first heterologous expression system developed in *P. chrysogenum* was to express a recombinant fungal xylanase gene and the cDNA for human tear lipocalin (Graessle et al. 1997). Since then, *P. chrysogenum* has been successfully used as expression system for protein production applying inducible, repressible, or constitutive promoters (Díez et al. 1999; Huber et al. 2019) and efforts were undertaken to define new promoters for strain engineering in this species (Polli et al. 2016). Recently two expression cassettes for homologous and heterologous gene expression were established in this fungus, resulting in an increase in the production of small, cysteine-rich, and cationic antifungal proteins with purification yields up to 80 mg/l (Sonderegger et al. 2016; Garrigues et al. 2018; Gandía et al. 2022).

*Penicillium oxalicum* has powerful protein secretion capability and has been applied for commercial cellulase production for many years (Fang et al. 2010). This fungus has been found to have relatively high homologous recombination frequencies in targeted gene manipulations (Li et al. 2010). After genetic engineering of the *P. oxalicum* 114-2, for instance, cellulose productivity of 158.38 U/l/h was reached (Han et al. 2017). Furthermore, an *A.* *niger* β-glucosidase-encoding gene was heterologously overexpressed in *P.* *oxalicum*. The resulted strain *P. oxalicum* C3-1 showed 156.6- and 245.2-fold increased production of the corresponding enzyme in the parent strain *P. oxalicum* PT3-1 and the wild-type strain *P. oxalicum* 114-2, respectively (Wang et al. 2019).

*Penicillium funiculosum* is a non-model hypercellulolytic fungus that produces high-quality protein mixtures for lignocellulosic biomass saccharification (Ogunmolu et al. 2015). This fungus is non-pathogenic, contains a diversity of extracellular enzyme-encoding genes, and is genetically manipulatable (Ogunmolu et al. 2018; Randhawa et al. 2021). To broaden the commercial potential of *P. funiculosum* as a host for the production of heterologous proteins, the promoter of the histone H4.1 gene was effectively exploited to drive the production of an intracellular bacterial enzyme, β-glucuronidase (GUS) (Belshaw et al. 2002).

*Penicillium* *verruculosum* is an efficient cellulase producer, making this species a good host for the production of heterologous proteins (Dotsenko et al. 2015). The expression of *A.* *niger* β-glucosidase gene under the control of either a strong cellobiohydrolase I (*cbh1*) gene promoter or a weaker histone (*hist4*) gene promoter provided notable boosting effect in *P. verruculosum* (Dotsenko et al. 2015)*.* Subsequently, the BGL and lytic polysaccharide monooxygenases (LPMOs) from *T.* *reesei* were produced by *P*. *verruculosum* B1-537 strain (Bulakhov et al. 2017). *P. verruculosum* was also used to produce heterologous endo-xanthanase (Denisenko et al. 2021).

*Penicillium canescens* exhibits an increased capacity for synthesizing secreted *β*-galactosidase and xylanase (Vavilova et al. 2003), and has also been established as a host for the production of heterologous proteins (Sinitsyn and Rozhkova 2015). As examples to illustrate the possibilities of *P. canescens* for the production of high value heterologous proteins, the *cbh1*, *cbh2*, and *egl2* genes encoding cellobiohydrolase I, cellobiohydrolase II, and endo-1,4-β-glucanase from *P. verruculosum* and *bglA* gene encoding the BGL from *A. niger* have been expressed in *P. canescens*, as well as the *inu1* and *inuA* genes from *Aspergillus* sp. encoding exo- and endo-inulinases (Sinitsyn and Rozhkova 2015).

Finally, *Penicillium subrubescens* is such a species that has a specific expansion of certain enzyme families related to hemicellulose, pectin, and inulin degradation (Mansouri et al. 2013; Peng et al. 2017). It has previously been demonstrated to be a potential industrial species and authors have established genome editing methodologies (Salazar-Cerezo et al. 2020). Thus, it is a promising new fungal cell factory for the production of heterologous proteins.

### *Myceliophthora*

*Myceliophthora thermophila* (synonym: *Sporotrichum thermophile*, previously known as *Chrysosporium lucknowense*) was isolated from the alkaline soil of eastern Russia and has excellent acid-base and temperature tolerance (Visser et al. 2011). With the ability to degrade plant biomass, *M. thermophila* provides a potentially rich reservoir of new enzymes for industrial uses, including numerous thermostable enzymes for biomass degradation (Gu et al. 2018). This thermophilic fungus has been developed into a mature system for carbohydrate hydrolase production at the industrial level, and the cellulase product from this fungus has also been granted GRAS status (Visser et al. 2011). The properties of *M. thermophila* provide an alternative for traditional fungal protein production hosts. For instance, a human IgG antibody against tumor necrosis factor alpha was expressed under control of the *cbh1* promoter in *M. thermophila*. Heterologous overexpression of fumarase gene from *Candida* *krusei* increased fumarate synthesis by up to 3-fold in *M.* *thermophila* (Gu et al. 2018). Disruption of protease genes in *M. thermophila* could at least double enhance the cellulase production, and the lower-protease producer could be used to further improve heterologous protein production (Li et al. 2020). *M. thermophila* is used to heterologously overproduce two different proteins, glucoamylase from *Myceliophthora heterothallica* and green fluorescent protein (Li et al. 2020).

*M.* *heterothallica* is a mostly unknown host organism with the ability to use sexual mating in strain development. In appearance, physiology, and phylogeny, the thermophile *M. heterothallica* is extremely similar to *M. thermophila* (Visser et al. 2011). The most notable distinction between these two species is the functional mating cycle of *M. heterothallica* (Aguilar-Pontes et al. 2016). In research, heterologous xylanase from *A.* *niger* was generated in *M. heterothallica*, resulting in an 80-fold increase in xylanase activity 24 h after sucrose induction. Therefore, *M. heterothallica* has potential as a host organism for heterologous protein production (Corinne 2016).

### Alternative fungal cell factories

Although many fungal hosts have already been used to produce proteins, the optimal host is largely unpredictable. Other filamentous fungi different from the aforementioned genera are also being studied in the laboratory to be identified as acceptable hosts for heterologous protein production. Several promising examples are listed in Table 1.

**Table 1:** Heterologous protein production in alternative potential fungal hosts

Expression host	Protein source	Protein	Yield	Reference
*Ashbya gossypii*	*Trichoderma reesei*	Endoglucanase I	440 g l^−1^	(Ribeiro et al. 2013)
*Acremonium chrysogenum*	Human	Thrombomodulin mutant protein	10 mg l^−1^	(Honda et al. 1997)
*Phanerochaete chrysosporium*	*Dichomitus squalens*	Thermostable manganese peroxidase	1.5 mg l^−1^	(Li et al. 2001)
*Phanerochaete sordida*	*Pleurocybella porrigens*	Lectin	0.2 mg l^−1^	(Suzuki et al. 2014)
*Talaromyces cellulolyticus*	*Pyrococcus* sp.	Endo-type cellulase EGPh and EGPf	0.63 g l^−1^ and 0.80 g l^−1^	(Kishishita et al. 2015)

## Strategies for heterologous protein production in filamentous fungi

In order to improve protein production in filamentous fungi, strategies such as using strong regulatory sequences (mainly promoters), codon optimization, replacing native signal peptides with more efficient ones, using carrier proteins, engineering glycosylation sites, regulation of unfolded protein response (UPR) and endoplasmic reticulum associated protein degradation (ERAD), optimization of the intracellular transport process, regulation of unconventional protein secretion (UPS), and construction of protease-deficient strains have been developed. These strategies are reviewed below.

### Use of different promoter sequences

Promoters are generally defined as the DNA sequence that is located upstream of the transcription start site, controlling the transcription of genes. A robust promoter element is essential for the production of heterologous proteins. Typically, promoters are divided into two types: constitutive and inducible (Jeennor et al. 2022). Constitutive promoters are always expressed regardless of environmental/culture conditions, whereas inducible promoters are activated in response to external biotic or abiotic triggers. Constitutive or inducible promoters may be preferable depending on the goal of gene expression.

Well-known constitutive promoters available for heterologous protein production in filamentous fungi include, e.g., promoters of the alcohol dehydrogenase gene (*adhA*) (Upshall et al. 1987), the small basic protein of unknown function and high expression gene (*cDNA1*) (Wohlschlager et al. 2021), the glyceraldehyde-3-phosphate dehydrogenase (*gpdA*) gene (Long et al. 2018), the pyruvate decarboxylase-encoding gene (*pdc1*) (Wang et al. 2013), and the manganese superoxide dismutase-encoding gene (*sodM*) (Wakai et al. 2014). Examples of enzymes produced with these and other constitutive promoters as well as the production yields achieved are shown in Table 2.

**Table 2:** Summary of constitutive and inducible promoters used in filamentous fungi for heterologous protein production

Protein (source)	Promoter* (origin)	Inducer/repressor	Host	Yield	Reference
Strong/constitutive promoters					
Antifungal protein PAF (*P. chrysogenum*)	*paf* (*P. chrysogenum)*	–/–	*P. digitatum*	83 mg l^−1^	(Sonderegger et al. 2016)
Antifungal protein NFAP (*Neosartorya fischeri*)	*paf* (*P. chrysogenum)*	–/–	*P. chrysogenum*	3 mg l^−1^	(Sonderegger et al. 2016)
Anti-MUC1 scFv antibody (human)	*ccg1nr* (*N. crassa*)	–/–	*N. crassa*	3 mg l^−1^	(Havlik et al. 2017)
Cellobiose dehydrogenase (*Corynascus thermophilus*)	*cDNA1* (*T. reesei*)	–/–	*T. reesei*	29 mg l^−1^	(Ma et al. 2017)
Class I hydrophobin DewA (*A. nidulans*)	*hfb2* (*T. reesei*)	–/–	*T. reesei*	33 mg l^−1^	(Schmoll et al. 2010)
Endoxylanase (*P. oxalicum*)	*pdc1* (*T. reesei*)	–/–	*T. reesei*	2 g l^−1^	(Wang et al. 2013)
Erythropoietin (human)	*gpdA* (*A. nidulans*)	–/–	*A. niger*	73.9 mg l^−1^	(Rojas-Sánchez et al. 2020)
Glyoxal oxidase (*Phanerochaete chrysosporium*)	*cDNA1* (*T. reesei*)	–/–	*T. reesei*	0.44 g l^−1^	(Wohlschlager et al. 2021)
Mannanase (*Trichoderma harzianum*)	*pdc1* (*T. reesei*)	–/–	*T. reesei*	1.6 g l^−1^	(Wang et al. 2014)
RNase A (bovine)	*cfp* (*N. crassa*)	–/–	*N. crassa*	356 mg l^−1^	(Allgaier et al. 2010)
Thaumatin (*Thaumatococcus daniellii*)	*gpdA* (*S. cerevisiae*)	–/–	*A. oryzae*	50 μg l^−1^	(Masuda and Kitabatake 2006)
Thaumatin (*T. daniellii*)	*gpdA* (*A. nidulans*)	–/–	*P. roqueforti*	2 mg l^−1^	(Masuda and Kitabatake 2006)
Tissue plasminogen activator (human)	*adhA* (*A. nidulans*)	–/–	*A. nidulans*	1 mg l^−1^	(Upshall et al. 1987)
Tissue plasminogen activator (human)	*tpiA* (*A. nidulans*)	–/–	*A. nidulans*	100 μg l^−1^	(Upshall et al. 1987)
Variable heavy-chain antibody (llama)	*sodM* (*A. oryzae*)	–/–	*A. oryzae*	73.8 mg l^−1^	(Okazaki et al. 2012)
Inducible promoters					
BGL I (*Aspergillus aculeatus*)	*cbh1* (*T. reesei*)	Various saccharides/glucose	*T. reesei*	15.1 g l^−1^	(Nakazawa et al. 2016)
BGL I (*A. aculeatus*)	*eg1* (*T. reesei*)	Various saccharides/glucose	*T. reesei*	17.4 g l^−1^	(Shibata et al. 2021)
BGL I (*A. aculeatus*)	*xyn3* (*T. reesei*)	Xylose	*T. reesei*	14.7 g l^−1^	(Shibata et al. 2021)
CBH I (*Melanocarpus albomyces*)	*cbh1* (*T. reesei*)	Various saccharides/glucose	*T. reesei*	4.9 g l^−1^	(Haakana et al. 2004)
CBH II (*M. albomyces*)	*cbh1* (*T. reesei*)	Various saccharides/glucose	*T. reesei*	4.3 g l^−1^	(Haakana et al. 2004)
CHY (bovine)	*amyB* (*A. oryzae*)	Starch or maltose/glucose	*A. oryzae*	109.4 mg l^−1^	(Yoon et al. 2011)
CHY (bovine)	*thiA* (*A. oryzae*)	Thiamine	*A. oryzae*	80 mg l^−1^	(Yoon et al. 2013)
Endoglucanase I (*M. albomyces*)	*cbh1* (*T. reesei*)	Various saccharides/glucose	*T. reesei*	7.4 g l^−1^	(Haakana et al. 2004)
HLY (human)	*amyB* (*A. oryzae*)	Starch or maltose/glucose	*A. oryzae*	35.8 mg l^−1^	(Yoon et al. 2011)
Interferon alpha-2b (human)	*cbh1* (*T. reesei*)	Various saccharides/glucose	*T. reesei*	4.5 g l^−1^	(Landowski et al. 2016)
Lactoferrin (human)	*amyA* (*A. oryzae*)	Starch or maltose/glucose	*A. oryzae*	25 mg l^−1^	(Ward et al. 1992)
Thaumatin (*T. daniellii*)	*glaA* (*A. niger*)	Glucose/xylose	*P. roqueforti*	2 mg l^−1^	(Masuda and Kitabatake 2006)
Tissue plasminogen activator (human)	*glaA* (*A. niger*)	Glucose/xylose	*A. niger*	25 mg l^−1^	(Wiebe et al. 2001)
Xylanase XYN2 (*Humicola grisea*)	*cbh1* (*T. reesei*)	Various saccharides/glucose	*T. reesei*	0.5 g l^−1^	(De Faria et al. 2002)
Xylanase XYN6 (*Acrophialophora nainiana*)	*cbh1* (*T. reesei*)	Various saccharides/glucose	*T. reesei*	172 mg l^−1^	(Salles et al. 2007)
Xylanase XYN11A (*Nonomuraea flexuosa*)	*cbh1* (*T. reesei*)	Various saccharides/glucose	*T. reesei*	820 mg l^−1^	(Paloheimo et al. 2007)
Xylanase XYNB (*Dictyoglomus thermophilum*)	*cbh1* (*T. reesei*)	Various saccharides/glucose	*T. reesei*	1 g l^−1^	(Nevalainen et al. 2018)
Xylanase XYNE (*P. canescens*)	*gla1* (*P. verruculosum*)	Glucose/xylose	*P. verruculosum*	29.3 g l^−1^	(Sinitsyn et al. 2018)

^*^Abbreviations: *adhA*, alcohol dehydrogenase; *amyA*, Taka-amylase; *amyB*, α-amylase; *cbh1*, cellobiohydrolase I; *ccg1nr*, a variant of glucose-repressible gene 1; *cDNA1*, small basic protein of unknown function and high expression; *cfp*, pyruvate decarboxylase; *egl1*, endoglucanases I; *gla1*, glucoamylase; *glaA*, glucoamylase A; *gpdA*, glyceraldehyde-3-phosphate dehydrogenase; *hfb2*, class II hydrophobin; *paf*, antifungal protein; *pdc1*, pyruvate decarboxylase; *sodM*, manganese superoxide dismutase; *tpiA*, triosephosphate isomerase; *thiA*, thiamine; *xyn3*, xylanase III

The main disadvantage of utilizing constitutively active gene promoters is that they are active during growth, leading to production of heterologous proteins that may be harmful to the host cells from the very beginning. As alternative, well-known inducible/repressible promoters have been used in many applications, including those from the Taka-amylase gene (*amyA*) (Okazaki et al. 2012), the cellobiohydrolase I (*cbh1*) (Long et al. 2018), the glucoamylase A gene (*glaA*) (Uchima et al. 2011), the thiamine biosynthesis-related gene (*thiA*) (Yoon et al. 2013), and the xylanase III gene (*xyn3*) (Rahman et al. 2009), as well as several others (Kluge et al. 2018; Sakekar et al. 2021). Examples of enzymes produced with these and other inducible promoters as well as the production yields achieved are shown in Table 2.

### Codon optimization of the heterologous genes

Codon optimization of heterologous genes is one of the most valuable tools for improving the production level of their corresponding proteins in filamentous fungi. Several studies have found that optimizing codons increases the steady-state mRNA level of heterologous genes (Tanaka et al. 2014; Fu et al. 2020). Most of the codon optimization strategies rely on using more frequently observed codons instead of rarely observed ones, which can increase the efficiency of the translation and the level of expression (Sen et al. 2020). The protein and mRNA production level of Der f7, a secreted protein of the house dust mite *Dermatophagoides farinae*, was increased by codon optimization in *A. oryzae* (Tokuoka et al. 2008). Tanaka et al. further demonstrated that a codon-optimized mite allergen gene *Der f7* increased the level of transcription and translation, as well as mRNA stability (Tanaka et al. 2012). Codon optimization of xylanase gene *xynB* from the thermophilic bacterium *Dictyoglomus thermophilum* allowed its expression in the filamentous fungus *T. reesei* by changing 20 codons (Te’o et al. 2000). In addition, it has been hypothesized that codon usage bias improves translation efficiency by accelerating elongation in highly expressed genes (Qian et al. 2012). Overall, optimal codons enhance the elongation rate, whereas non-optimal codons reduce it (Yu et al. 2015).

### Insertion of other signal peptides

Signal peptides are short peptides located in the N-terminal of proteins and are responsible for transport of proteins to and through the endoplasmic reticulum (ER) and the secretory pathway. Consequently, signal peptides are very important for the secretion of the recombinant proteins, as they facilitate the purification processes (Owji et al. 2018). Replacing the original signal peptide with a more efficient one tends to increase the secretion efficiency of heterologous proteins. In *A.* *niger*, by replacing the native signal peptide of an α-galactosidase with the GlaA signal peptide, the extracellular α-galactosidase activity increased by more than 22 times (Xu et al. 2018). Similarly, by using this approach, the thermostable trehalase gene from *M. thermophila* has also been successfully expressed in *A.* *niger* (Dong et al. 2020). Also, the use of heterologous signal peptides can result in high levels of recombinant protein secretion. For example, in the phytopathogenic fungus *Penicillium digitatum*, the signal peptide from the *P. chrysogenum* antifungal protein PAF allowed the production and secretion of antifungal proteins in this phytopathogen for the first time in high yields (Garrigues et al. 2017).

### Insertion of introns in heterologous genes

Introns as regulatory elements can boost gene expression without functioning as binding sites for transcription factors. This phenomenon was called “intron-mediated enhancement” (Gallegos and Rose 2015). Introns can enhance transcript levels by affecting transcription rate, nuclear export, and transcript stability. Furthermore, introns can improve mRNA translation efficiency (Shaul 2017). There are several reports about the enhancement of gene expression in mammals, plants, yeasts, and insects (Shaul 2017; Baier et al. 2018; Emami et al. 2013), but knowledge in filamentous fungi is sparse. A strategy was tried to improve the heterologous protein production by incorporating introns into the open reading frame of a given gene. Xu and Gong demonstrated that introns play crucial roles in the antifungal protein gene expression from *Aspergillus giganteus* in *T. viride* by affecting mRNA accumulation (Xu and Gong 2003). The level of expression of human erythropoietin was enhanced in *A. niger* by incorporating the introns from the D-fructose-1,6-bisphosphatase encoding gene of *A. niger* into the erythropoietin sequence (Rojas-Sánchez et al. 2020).

## Development of protein production systems

### Carrier proteins to enhance production of heterologous proteins

The genetic fusion of a target protein with a native, well-secreted protein known as “carrier” is a commonly used strategy to increase heterologous protein production in filamentous fungi (Hoang et al. 2015). This approach appears to improve mRNA stability, promote translocation in the secretory pathway, and prevent protein from degradation (Ntana et al. 2020). The GlaA, α-amylase, and CbhI proteins from *A.* *niger*, *A. oryzae*, and *T. reesei*, respectively, have been used as carriers to produce heterologous proteins. With this approach, the production of heterologous proteins was increased by 5- to 1000-fold, depending on the strain and protein, resulting in yields ranging from 1 to 2000 mg/l (James et al. 2012). For instance, the in-frame fusion of human protein granulocyte colony stimulating factor with the native GlaA allowed production of 5–10 mg/l of this growth factor in *A.* *niger* (Wang et al. 2020). When CHY was fused to the native α-amylase in *A. oryzae*, the fused CHY produced twice as much as the non-fused CHY (Ohno et al. 2011).

However, releasing the heterologous protein from the carrier protein may be a bottleneck. When the production of human interferon alpha-2b in *T. reesei* was fused toe CbhI as a carrier protein, 44% of the fusion protein could not be cleaved by the protease Kex2 (Landowski et al. 2016).

### Influence of glycosylation

Protein glycosylation is the most common form of posttranslational modification on excreted and extracellular membrane-associated proteins (Spiro 2002). It involves the covalent attachment of many different types of glycans to a protein. Modifying protein glycosylation can be another possible strategy for increasing the stability and production of heterologous proteins in fungi. For example, N-glycosylation of *A. terreus* BGL, which was heterologously produced in *T. reesei*, positively affected stability of the resulting protein (Wei et al. 2013). In addition, it has been reported that removing the N384 glycosylation site of the CbhI during its production in *A.* *niger* resulted in a mutant strain with 70% higher cellulase activity (Adney et al. 2009). van den Brink et al. (2006) improved a poorly used N-glycosylation site within the prochymosin molecule, and the resulting highly glycosylated chymosin was produced in *A. niger* with a yield increase of more than 100% compared to the native molecule. In conclusion, even though some examples of protein glycosylation modification have shown improved protein production, in some cases, adding or removing glycosylation may change the enzyme properties, and therefore, experiments to further address enzyme functionality are required.

### Regulation of the UPR and ERAD to promote protein secretion

Protein secretion, and in particular protein folding and maturation, takes place through the endoplasmic reticulum (ER). Overproduction of homologous or heterologous proteins could disturb the balance of ER and further cause the UPR and ERAD processes to relieve the stress in filamentous fungi (Heimel 2015). Therefore, attempts to improve heterologous protein production in filamentous fungi mostly focus on the secretory pathway and the folding of proteins in the ER (Sun and Su 2019). For example, overexpression of the *hac1* and *bip1* genes separately, which are involved in UPR, was found to enhance secretion of the *A. niger* glucose oxidase in *T. reesei* by 1.5- and 1.8-fold (Wu et al. 2017). In another study, deletion of the *derA* and *hrdC* genes, which are involved in the ERAD pathway, resulted in 6-fold and 2-fold increase in the heterologous β-glucuronidase in *A.* *niger*, respectively (Carvalho et al. 2011). The beneficial impact of downregulating ERAD components might be attributable to the increased time for proteins to be (re)folded in the ER before being targeted for degradation. However, loss of ERAD components increases UPR activation, which is most likely caused by an accumulation of un- or misfolded proteins in the ER, and can negatively influence growth behavior (Carvalho et al. 2011; Krishnan et al. 2013).

### Optimization of the intracellular transport process

Optimization of the intracellular transport process results in enhanced secretion of heterologous proteins (Wang et al. 2020). By genetically deleting the putative ER-Golgi cargo receptors AoVip36 and AoEmp47, which can hinder the secretion of heterologous proteins by promoting their retention in the ER, the production of the α-amylase-fused form of bovine prochymosin was increased by approximately 2-fold in *A. oryzae* (Hoang et al. 2015). Overexpression of the *snc1* gene, which is involved in the fusion of vesicles and plasma membrane, was reported to increase by 2.2-fold the production of an *A.* *niger* glucose oxidase in *T. reesei* (Wu et al. 2017).

In addition, vacuolar protein sorting (VPS) also affects the secretion of heterologous proteins in filamentous fungi. An interesting observation was that disruption of the VPS receptor gene *Aovps10* in *A. oryzae* enhanced the production of CHY and HLY by 3- and 2.2-fold (Yoon et al. 2010). Furthermore, autophagy was reported to deliver misfolded secretory proteins accumulated in the ER to vacuoles, which is an important process that adversely affects heterologous protein production (Kimura et al. 2011), since heterologous proteins are often recognized as misfolded proteins. The authors described that disrupting several autophagy genes in *A. oryzae* enhanced the production levels of CHY up to 3-fold compared to the parental strain (Yoon et al. 2013).

### Unconventional secretion for production of heterologous proteins

Most of the extracellular proteins are secreted by the classical ER/Golgi-dependent pathway. However, some extracellular proteins can be secreted without passing through the conventional secretion pathway and are transported to the cell membrane via alternative routes, which are known as UPS pathways (Krombach et al. 2018). In filamentous fungi, unconventional secretion has been observed for endochitinase Cst1 from *Ustilago* *maydis* (Stock et al. 2012) as well as for the aspartic protease from *A.* *niger* (Burggraaf et al. 2016). It has been reported that the use of this pathway for the production of heterologous proteins could result in improved production yields and activities, as the proteins produced via UPS most likely do not undergo unwanted post-translational modifications. Exploiting the bacterial enzyme β-glucuronidase (GUS) as a reporter for unconventional secretion in *U. maydis*, the yields of Gus-Cst1 fusion protein were increased by a combination of culture buffering and deletion of harmful proteases (Terfrüchte et al. 2018). Furthermore, using the kinase Don3 as a gatekeeper to control the UPS was successfully established in *U. maydis* (Hussnaetter et al. 2021).

### Construction of a protease-deficient strain

Proteolytic degradation of the products has been thought to be one of the bottlenecks limiting yields of heterologous protein production in filamentous fungi (Qian et al. 2019). Discovering that extracellular proteases can decrease the amounts of heterologous proteins has led to the development of protease-deficient strains. Deletion of protease-encoding genes is one of the methods to establish protease-deficient strains. In *A. oryzae*, several protease-encoding genes were disrupted to improve the production of CHY and HLY (Jin et al. 2007; Maruyama and Kitamoto 2008; Yoon et al. 2011; Kitamoto et al. 2015). In *T. reesei*, the production levels of human interferon alpha-2b were increased upon deletion of the subtilisin protease gene *slp7* and the metalloprotease gene *amp2*, with yields of 2.1 g/l and 2.4 g/l, respectively (Landowski et al. 2016). To improve the laccase yield, two genes encoding major proteolytic activities, dipeptidyl-peptidase and aspartic protease, were successfully disrupted in *A. nidulans*, and the activity of the *Pycnoporus* *sanguineus* laccase was increased approximately 13-fold (Li et al. 2018).

Disruption of protease regulatory genes has also been effective in substantially reducing protease activity in many filamentous fungi. For instance, the deletion of the transcription factor PrtT resulted in the reduction of major extracellular proteases in *A.* *niger*, and the production of *Glomerella cingulata* cutinase was increased 36-fold compared to that of the parental strain (Kamaruddin et al. 2017). Deletion of the p53-like transcriptional factor Vib1 in *T. reesei* exhibited a drastic decrease in cellulase and protease secretion, and the *A. niger* BGL encoding gene *bglA* was expressed at higher levels in Δ*vib1* strain (Sun et al. 2022). In another study, the *T. reesei* engineered platform was constructed by deleting eleven major lignocellulose-degrading enzymes and/or the putative protease transcription factor Pea1. Subsequently, the levels of production of three heterologous proteins, bacterial xylanase, fungal immunomodulatory proteins, and human serum albumin, were increased in *T. reesei* under the control of *cbh1* promoter (Chai et al. 2022).

## Conclusions and future prospects

Owing to the high efficiency of protein secretion and excellent capability of posttranslational modification, filamentous fungi are considered as promising cell factories for heterologous protein expression and secretion (Sakekar et al. 2021). However, the expression systems for heterologous genes in filamentous fungi are still underdeveloped due to a lack of profound comprehension of their regulation of gene expression and protein secretory pathways (Ntana et al. 2020). The different approaches that could improve heterologous gene expression in filamentous fungi are elaborated in this review. While the complexity of these multiple methods seems challenging, they provide opportunities to conduct optimization experiments for heterologous protein production and to expand the knowledge on protein secretory pathways and processes in filamentous fungi. The further development of transcriptomics, proteomics, and metabolomics will provide more in depth understanding of protein production in filamentous fungi and will enable the development of more efficient fungal cell factories.

## References

[CR1] Adney WS, Jeoh T, Beckham GT, Chou YC, Baker JO, Michener W, Brunecky R, Himmel ME (2009). Probing the role of N-linked glycans in the stability and activity of fungal cellobiohydrolases by mutational analysis. Cellulose.

[CR2] Aguilar-Pontes MV, Zhou M, van der Horst S, Theelen B, de Vries RP, van den Brink J (2016). Sexual crossing of thermophilic fungus *Myceliophthora heterothallica* improved enzymatic degradation of sugar beet pulp. Biotechnol Biofuels.

[CR3] Alberto F, Navarro D, de Vries RP, Asther M, Record E (2009). Technical advance in fungal biotechnology: development of a miniaturized culture method and an automated high-throughput screening. Lett Appl Microbiol.

[CR4] Allgaier S, Weiland N, Hamad I, Kempken F (2010). Expression of ribonuclease A and ribonuclease N1 in the filamentous fungus *Neurospora crassa*. Appl Microbiol Biotechnol.

[CR5] Baier T, Wichmann J, Kruse O, Lauersen KJ (2018). Intron-containing algal transgenes mediate efficient recombinant gene expression in the green microalga *Chlamydomonas reinhardtii*. Nucleic Acids Res.

[CR6] Belshaw NJ, Haigh NP, Fish NM, Archer DB, Alcocer MJC (2002). Use of a histone H4 promoter to drive the expression of homologous and heterologous proteins by *Penicillium funiculosum*. Appl Microbiol Biotechnol.

[CR7] Bulakhov AG, Volkov PV, Rozhkova AM, Gusakov AV, Nemashkalov VA, Satrutdinov AD, Sinitsyn AP (2017). Using an inducible promoter of a gene encoding *Penicillium verruculosum* glucoamylase for production of enzyme preparations with enhanced cellulase performance. PLoS One.

[CR8] Burggraaf AM, Punt PJ, Ram AFJ (2016). The unconventional secretion of PepN is independent of a functional autophagy machinery in the filamentous fungus *Aspergillus niger*. FEMS Microbiol Lett.

[CR9] Cairns TC, Nai C, Meyer V (2018). How a fungus shapes biotechnology: 100 years of *Aspergillus niger* research. Fungal Biol Biotechnol.

[CR10] Carvalho NDSP, Arentshorst M, Kooistra R, Stam H, Sagt CM, van den Hondel CAMJJ, Ram AFJ (2011). Effects of a defective ERAD pathway on growth and heterologous protein production in *Aspergillus niger*. Appl Microbiol Biotechnol.

[CR11] Chai S, Zhu Z, Tian E, Xiao M, Wang Y, Zou G, Zhou Z (2022). Building a versatile protein production platform using engineered *Trichoderma reesei*. ACS Synth Biol.

[CR12] Chen X, Wang B, Pan L (2019). Heterologous expression and characterization of *Penicillium citrinum* nuclease P1 in *Aspergillus niger* and its application in the production of nucleotides. Protein Expr Purif.

[CR13] Corinne D (2016) The use of filamentous fungi *Myceliophthora heterothallica* as a host for heterologous protein production. MSc Thesis, Concordia University, Montreal, Canada. https://spectrum.library.concordia.ca/id/eprint/981446/

[CR14] Culleton H, Bouzid O, McKie VA, de Vries RP (2014). New promoters to improve heterologous protein production in *Aspergillus vadensis*. Curr Biotechnol.

[CR15] Daba GM, Mostafa FA, Elkhateeb WA (2021). The ancient koji mold (*Aspergillus oryzae*) as a modern biotechnological tool. Bioresour Bioprocess.

[CR16] De Faria FP, Te’O VSJ, Bergquist PL, Azevedo MO, Nevalainen KMH (2002). Expression and processing of a major xylanase (XYN2) from the thermophilic fungus *Humicola grisea* var. *thermoidea* in *Trichoderma reesei*. Lett Appl Microbiol.

[CR17] de Vries RP, Burgers K, van de Vondervoort PJI, Frisvad JC, Samson RA, Visser J (2004). A new black *Aspergillus* species, *A. vadensis*, is a promising host for homologous and heterologous protein production. Appl Environ Microbiol.

[CR18] de Vries RP, Frisvad JC, van de Vondervoort PJI, Burgers K, Kuijpers AFA, Samson RA, Visser J (2005). *Aspergillus vadensis*, a new species of the group of black aspergilli. Antonie Van Leeuwenhoek.

[CR19] Denisenko YA, Korotkova OG, Zorov IN, Rozhkova AM, Semenova MV, Elcheninov AG, Kublanov IV, Sinitsyn AP (2021). Heterologous expression of *Thermogutta terrifontis* endo-xanthanase in *Penicillium verruculosum*, isolation and primary characterization of the enzyme. Biochemistry.

[CR20] Díez B, Mellado E, Rodríguez M, Bernasconi E, Barredo JL (1999). The NADP-dependent glutamate dehydrogenase gene from *Penicillium chrysogenum* and the construction of expression vectors for filamentous fungi. Appl Microbiol Biotechnol.

[CR21] Dong L, Lin X, Yu D, Huang L, Wang B, Pan L (2020). High-level expression of highly active and thermostable trehalase from *Myceliophthora thermophila* in *Aspergillus niger* by using the CRISPR/Cas9 tool and its application in ethanol fermentation. J Ind Microbiol Biotechnol.

[CR22] Dotsenko GS, Gusakov AV, Rozhkova AM, Korotkova OG, Sinitsyn AP (2015). Heterologous β-glucosidase in a fungal cellulase system: comparison of different methods for development of multienzyme cocktails. Process Biochem.

[CR23] Duruksu G, Ozturk B, Biely P, Bakir U, Ogel ZB (2009). Cloning, expression and characterization of endo-β-1,4-mannanase from *Aspergillus fumigatus* in *Aspergillus sojae* and *Pichia pastoris*. Biotechnol Prog.

[CR24] Emami S, Arumainayagam D, Korf I, Rose AB (2013). The effects of a stimulating intron on the expression of heterologous genes in *Arabidopsis thaliana*. Plant Biotechnol J.

[CR25] Fang X, Shen Y, Zhao J, Bao X, Qu Y (2010). Status and prospect of lignocellulosic bioethanol production in China. Bioresour Technol.

[CR26] Fu H, Liang Y, Zhong X, Pan ZL, Huang L, Zhang HL, Xu Y, Zhou W, Liu Z (2020). Codon optimization with deep learning to enhance protein expression. Sci Rep.

[CR27] Gallegos JE, Rose AB (2015). The enduring mystery of intron-mediated enhancement. Plant Sci.

[CR28] Gandía M, Moreno-Giménez E, Giner-Llorca M, Garrigues S, Ropero-Pérez C, Locascio A, Martínez-Culebras PV, Marcos JF, Manzanares P (2022). Development of a FungalBraid *Penicillium expansum*-based expression system for the production of antifungal proteins in fungal biofactories. Microb Biotechnol.

[CR29] Garrigues S, Gandía M, Popa C, Borics A, Marx F, Coca M, Marcos JF, Manzanares P (2017). Efficient production and characterization of the novel and highly active antifungal protein AfpB from *Penicillium digitatum*. Sci Rep.

[CR30] Garrigues S, Gandía M, Castillo L, Coca M, Marx F, Marcos JF, Manzanares P (2018). Three antifungal proteins from *Penicillium expansum*: different patterns of production and antifungal activity. Front Microbiol.

[CR31] Gonçalves TA, Damásio ARL, Segato F, Alvarez TM, Bragatto J, Brenelli LB, Citadini APS, Murakami MT, Ruller R, Paes Leme AF, Prade RA, Squina FM (2012). Functional characterization and synergic action of fungal xylanase and arabinofuranosidase for production of xylooligosaccharides. Bioresour Technol.

[CR32] Graessle S, Haas H, Friedlin E, Kürnsteiner H, Stöffler G, Redl B (1997). Regulated system for heterologous gene expression in *Penicillium chrysogenum*. Appl Environ Microbiol.

[CR33] Gu S, Li J, Chen B, Sun T, Liu Q, Xiao D, Tian C (2018). Metabolic engineering of the thermophilic filamentous fungus *Myceliophthora thermophila* to produce fumaric acid. Biotechnol Biofuels.

[CR34] Gürkök S, Söyler B, Biely P, Ögel ZB (2010). Cloning and heterologous expression of the extracellular alpha-galactosidase from Aspergillus fumigatus in *Aspergillus sojae* under the control of *gpdA* promoter. J Mol Catal B Enzym.

[CR35] Haakana H, Miettinen-Oinonen A, Joutsjoki V, Mäntylä A, Suominen P, Vehmaanperä J (2004). Cloning of cellulase genes from *Melanocarpus albomyces* and their efficient expression in *Trichoderma reesei*. Enzyme Microb Technol.

[CR36] Haefner S, Knietsch A, Scholten E, Braun J, Lohscheidt M, Zelder O (2005). Biotechnological production and applications of phytases. Appl Microbiol Biotechnol.

[CR37] Han X, Song W, Liu G, Li Z, Yang P, Qu Y (2017). Improving cellulase productivity of *Penicillium oxalicum* RE-10 by repeated fed-batch fermentation strategy. Bioresour Technol.

[CR38] Harkki A, Uusitalo J, Bailey M, Penttila M, Knowles JKC (1989). A novel fungal expression system: secretion of active calf chymosin from the filamentous fungus *Trichoderma reesei*. Nat Biotechnol.

[CR39] Havlik D, Brandt U, Bohle K, Fleißner A (2017). Establishment of *Neurospora crassa* as a host for heterologous protein production using a human antibody fragment as a model product. Microb Cell Fact.

[CR40] Heimel K (2015). Unfolded protein response in filamentous fungi—implications in biotechnology. Appl Microbiol Biotechnol.

[CR41] Hoang HD, Maruyama JI, Kitamoto K (2015). Modulating endoplasmic reticulum-Golgi cargo receptors for improving secretion of carrier-fused heterologous proteins in the filamentous fungus *Aspergillus oryzae*. Appl Environ Microbiol.

[CR42] Honda G, Matsuda A, Zushi M, Yamamoto S, Komatsu KI (1997). Heterologous protein production in *Acremonium chrysogenum*: expression of bacterial cephalosporin C acylase and human thrombomodulin genes. Biosci Biotechnol Biochem.

[CR43] Huber A, Lerchster H, Marx F (2019). Nutrient excess triggers the expression of the *Penicillium chrysogenum* antifungal protein PAFB. Microorganisms.

[CR44] Hussnaetter KP, Philipp M, Müntjes K, Feldbrügge M, Schipper K (2021). Controlling unconventional secretion for production of heterologous proteins in *Ustilago maydis* through transcriptional regulation and chemical inhibition of the kinase Don3. J Fungi.

[CR45] James ER, Van Zyl WH, Van Zyl PJ, Görgens JF (2012). Recombinant hepatitis B surface antigen production in *Aspergillus niger*: evaluating the strategy of gene fusion to native glucoamylase. Appl Microbiol Biotechnol.

[CR46] Jatuwong K, Suwannarach N, Kumla J, Penkhrue W, Kakumyan P, Lumyong S (2020). Bioprocess for production, characteristics, and biotechnological applications of fungal phytases. Front Microbiol.

[CR47] Jeennor S, Anantayanon J, Chutrakul C, Panchanawaporn S, Laoteng K (2022). Novel pentose-regulated promoter of *Aspergillus oryzae* with application in controlling heterologous gene expression. Biotechnol Rep.

[CR48] Jin FJ, Watanabe T, Juvvadi PR, Maruyama JI, Arioka M, Kitamoto K (2007). Double disruption of the proteinase genes, *tppA* and *pepE*, increases the production level of human lysozyme by *Aspergillus oryzae*. Appl Microbiol Biotechnol.

[CR49] Jin FJ, Hu S, Wang BT, Jin L (2021). Advances in genetic engineering technology and its application in the industrial fungus *Aspergillus oryzae*. Front Microbiol.

[CR50] Jørgensen MS, Skovlund DA, Johannesen PF, Mortensen UH (2014). A novel platform for heterologous gene expression in *Trichoderma reesei* (teleomorph *Hypocrea jecorina*). Microb Cell Fact.

[CR51] Kalra R, Conlan XA, Goel M (2020). Fungi as a potential source of pigments: harnessing filamentous fungi. Front Chem.

[CR52] Kamaruddin N, Storms R, Mahadi NM, Illias RM, Bakar FDA, Murad AMA (2017). Reduction of extracellular proteases increased activity and stability of heterologous protein in *Aspergillus niger*. Arab J Sci Eng.

[CR53] Kimura S, Maruyama J-i, Kikuma T, Arioka M, Kitamoto K (2011). Autophagy delivers misfolded secretory proteins accumulated in endoplasmic reticulum to vacuoles in the filamentous fungus *Aspergillus oryzae*. Biochem Biophys Res Commun.

[CR54] Kishishita S, Fujii T, Ishikawa K (2015). Heterologous expression of hyperthermophilic cellulases of archaea *Pyrococcus* sp. by fungus *Talaromyces cellulolyticus*. J Ind Microbiol Biotechnol.

[CR55] Kitamoto N, Ono N, Yoshino-Yasuda S (2015). Construction of quintuple protease and double amylase gene deletant for heterologous protein production in *Aspergillus oryzae* KBN616. Food Sci Technol Res.

[CR56] Kluge J, Terfehr D, Kück U (2018). Inducible promoters and functional genomic approaches for the genetic engineering of filamentous fungi. Appl Microbiol Biotechnol.

[CR57] Krishnan K, Feng X, Powers-Fletcher MV, Bick G, Richie DL, Woollett LA, Askew DS (2013). Effects of a defective endoplasmic reticulum-associated degradation pathway on the stress response, virulence, and antifungal drug susceptibility of the mold pathogen *Aspergillus fumigatus*. Eukaryot Cell.

[CR58] Krombach S, Reissmann S, Kreibich S, Bochen F, Kahmann R (2018). Virulence function of the *Ustilago maydis* sterol carrier protein 2. New Phytol.

[CR59] Kumar A (2020) *Aspergillus nidulans*: a potential resource of the production of the native and heterologous enzymes for industrial applications. Int J Microbiol 2020. 10.1155/2020/8894215 10.1155/2020/8894215 PMC7416255 32802076

[CR60] Landowski CP, Mustalahti E, Wahl R, Croute L, Sivasiddarthan D, Westerholm-Parvinen A, Sommer B, Ostermeier C, Helk B, Saarinen J, Saloheimo M (2016). Enabling low cost biopharmaceuticals: high level interferon alpha-2b production in *Trichoderma reesei*. Microb Cell Fact.

[CR61] Li D, Youngs HL, Gold MH (2001). Heterologous expression of a thermostable manganese peroxidase from *Dichomitus squalens* in *Phanerochaete chrysosporium*. Arch Biochem Biophys.

[CR62] Li ZH, Du CM, Zhong YH, Wang TH (2010). Development of a highly efficient gene targeting system allowing rapid genetic manipulations in *Penicillium decumbens*. Appl Microbiol Biotechnol.

[CR63] Li W, Yu J, Li Z, Yin WB (2018). Rational design for fungal laccase production in the model host *Aspergillus nidulans*. Sci China Life Sci.

[CR64] Li X, Liu Q, Sun W, He Q, Tian C (2020). Improving cellulases production by *Myceliophthora thermophila* through disruption of protease genes. Biotechnol Lett.

[CR65] Li Q, Lu J, Zhang G, Liu S, Zhou J, Du G, Chen J (2022). Recent advances in the development of *Aspergillus* for protein production. Bioresour Technol.

[CR66] Long L, Zhao H, Ding D, Xu M, Ding S (2018). Heterologous expression of two *Aspergillus niger* feruloyl esterases in *Trichoderma reesei* for the production of ferulic acid from wheat bran. Bioprocess Biosyst Eng.

[CR67] Lübeck M, Lübeck PS (2022). Fungal cell factories for efficient and sustainable production of proteins and peptides. Microorganisms.

[CR68] Ma S, Preims M, Piumi F, Kappel L, Seiboth B, Record E, Kracher D, Ludwig R (2017). Molecular and catalytic properties of fungal extracellular cellobiose dehydrogenase produced in prokaryotic and eukaryotic expression systems. Microb Cell Fact.

[CR69] Madhavan A, Pandey A, Sukumaran RK (2017). Expression system for heterologous protein expression in the filamentous fungus *Aspergillus unguis*. Bioresour Technol.

[CR70] Madhavan A, Arun KB, Sindhu R, Alphonsa Jose A, Pugazhendhi A, Binod P, Sirohi R, Reshmy R, Kumar Awasthi M (2022). Engineering interventions in industrial filamentous fungal cell factories for biomass valorization. Bioresour Technol.

[CR71] Mansouri S, Houbraken J, Samson RA, Frisvad JC, Christensen M, Tuthill DE, Koutaniemi S, Hatakka A, Lankinen P (2013). *Penicillium subrubescens*, a new species efficiently producing inulinase. Antonie Van Leeuwenhoek.

[CR72] Maruyama JI, Kitamoto K (2008). Multiple gene disruptions by marker recycling with highly efficient gene-targeting background (Δ*ligD*) in *Aspergillus oryzae*. Biotechnol Lett.

[CR73] Masuda T, Kitabatake N (2006). Developments in biotechnological production of sweet proteins. J Biosci Bioeng.

[CR74] Meng X, Fang Y, Ding M, Zhang Y, Jia K, Li Z, Collemare J, Liu W (2022). Developing fungal heterologous expression platforms to explore and improve the production of natural products from fungal biodiversity. Biotechnol Adv.

[CR75] Meyer V, Andersen MR, Brakhage AA, Braus GH, Caddick MX, Cairns TC, de Vries RP, Haarmann T, Hansen K, Hertz-fowler C, Krappmann S, Mortensen UH, Peñalva MA, Ram AFJ, Head RM (2016). Current challenges of research on filamentous fungi in relation to human welfare and a sustainable bio-economy: a white paper. Fungal Biol Biotechnol.

[CR76] Meyer V, Basenko EY, Benz JP, Braus GH, Caddick MX, Csukai M, de Vries RP, Endy D, Frisvad JC, Gunde-Cimerman N, Haarmann T, Hadar Y, Hansen K, Johnson RI, Keller NP, Kraševec N, Mortensen UH, Perez R, Ram AFJ, Record E, Ross P, Shapaval V, Steiniger C, Van Den Brink H, Van Munster J, Yarden O, Wösten HAB (2020). Growing a circular economy with fungal biotechnology: a white paper. Fungal Biol Biotechnol.

[CR77] Nakazawa H, Kawai T, Ida N, Shida Y, Shioya K, Kobayashi Y, Okada H, Tani S, Sumitani J-i, Kawaguchi T, Morikawa Y, Ogasawara W (2016). A high performance *Trichoderma reesei* strain that reveals the importance of xylanase III in cellulosic biomass conversion. Enzyme Microb Technol.

[CR78] Nevalainen H, Bergquist P, Te’o VSJ (2018). Making a bacterial thermophilic enzyme in a fungal expression system. Curr Protoc Protein Sci.

[CR79] Ntana F, Mortensen UH, Sarazin C, Figge R (2020). *Aspergillus*: a powerful protein production platform. Catalysts.

[CR80] Ogunmolu FE, Kaur I, Gupta M, Bashir Z, Pasari N, Yazdani SS (2015). Proteomics insights into the biomass hydrolysis potentials of a hypercellulolytic fungus *Penicillium funiculosum*. J Proteome Res.

[CR81] Ogunmolu FE, Kaur I, Pasari N, Gupta M, Yazdani SS (2018). Quantitative multiplexed profiling of *Penicillium funiculosum* secretome grown on polymeric cellulase inducers and glucose. J Proteomics.

[CR82] Ohno A, Maruyama JI, Nemoto T, Arioka M, Kitamoto K (2011). A carrier fusion significantly induces unfolded protein response in heterologous protein production by *Aspergillus oryzae*. Appl Microbiol Biotechnol.

[CR83] Okazaki F, Aoki JI, Tabuchi S, Tanaka T, Ogino C, Kondo A (2012). Efficient heterologous expression and secretion in *Aspergillus oryzae* of a llama variable heavy-chain antibody fragment VHH against EGFR. Appl Microbiol Biotechnol.

[CR84] Owji H, Nezafat N, Negahdaripour M, Hajiebrahimi A, Ghasemi Y (2018). A comprehensive review of signal peptides: structure, roles, and applications. Eur J Cell Biol.

[CR85] Paloheimo M, Mäntylä A, Kallio J, Puranen T, Suominen P (2007). Increased production of xylanase by expression of a truncated version of the *xyn11A* gene from *Nonomuraea flexuosa* in *Trichoderma reesei*. Appl Environ Microbiol.

[CR86] Peng M, Dilokpimol A, Mäkelä MR, Hildén K, Bervoets S, Riley R, Grigoriev IV, Hainaut M, Henrissat B, de Vries RP, Granchi Z (2017). The draft genome sequence of the ascomycete fungus *Penicillium subrubescens* reveals a highly enriched content of plant biomass related CAZymes compared to related fungi. J Biotechnol.

[CR87] Polli F, Meijrink B, Bovenberg RAL, Driessen AJM (2016). New promoters for strain engineering of *Penicillium chrysogenum*. Fungal Genet Biol.

[CR88] Qian W, Yang JR, Pearson NM, Maclean C, Zhang J (2012). Balanced codon usage optimizes eukaryotic translational efficiency. PLoS Genet.

[CR89] Qian Y, Zhong L, Sun Y, Sun N, Zhang L, Liu W, Qu Y, Zhong Y (2019). Enhancement of cellulase production in *Trichoderma reesei* via disruption of multiple protease genes identified by comparative secretomics. Front Microbiol.

[CR90] Rahman Z, Shida Y, Furukawa T, Suzuki Y, Okada H, Ogasawara W, Morikawa Y (2009). Evaluation and characterization of *Trichoderma reesei* cellulase and xylanase promoters. Appl Microbiol Biotechnol.

[CR91] Randhawa A, Pasari N, Sinha T, Gupta M, Nair AM, Ogunyewo OA, Verma S, Verma PK, Yazdani SS (2021). Blocking drug efflux mechanisms facilitate genome engineering process in hypercellulolytic fungus, *Penicillium funiculosum* NCIM1228. Biotechnol Biofuels.

[CR92] Rantasalo A, Vitikainen M, Paasikallio T, Jäntti J, Landowski CP, Mojzita D (2019). Novel genetic tools that enable highly pure protein production in *Trichoderma reesei*. Sci Rep.

[CR93] Ribeiro O, Magalhães F, Aguiar TQ, Wiebe MG, Penttilä M, Domingues L (2013). Random and direct mutagenesis to enhance protein secretion in *Ashbya gossypii*. Bioengineered.

[CR94] Rojas-Sánchez U, López-Calleja AC, Millán-Chiu BE, Fernández F, Loske AM, Gómez-Lim MA (2020). Enhancing the yield of human erythropoietin in *Aspergillus niger* by introns and CRISPR/Cas9. Protein Expr Purif.

[CR95] Romero SM, Giudicessi SL, Vitale RG (2021). Is the fungus *Aspergillus* a threat to cultural heritage?. J Cult Herit.

[CR96] Sakekar AA, Gaikwad SR, Punekar NS (2021). Protein expression and secretion by filamentous fungi. J Biosci.

[CR97] Salazar-Cerezo S, Kun RS, de Vries RP, Garrigues S (2020). CRISPR/Cas9 technology enables the development of the filamentous ascomycete fungus *Penicillium subrubescens* as a new industrial enzyme producer. Enzyme Microb Technol.

[CR98] Salles BC, Te’o VSJ, Gibbs MD, Bergquist PL, Filho EXF, Ximenes EA, Nevalainen KMH (2007). Identification of two novel xylanase-encoding genes (*xyn5* and *xyn6*) from *Acrophialophora nainiana* and heterologous expression of *xyn6* in *Trichoderma reesei*. Biotechnol Lett.

[CR99] Schmoll M, Seibel C, Kotlowski C, Wöllert Genannt Vendt F, Liebmann B, Kubicek CP (2010). Recombinant production of an *Aspergillus nidulans* class i hydrophobin (DewA) in *Hypocrea jecorina* (*Trichoderma reesei*) is promoter-dependent. Appl Microbiol Biotechnol.

[CR100] Sen A, Kargar K, Akgün E, Plnar MC (2020). Codon optimization: a mathematical programing approach. Bioinformatics.

[CR101] Shaul O (2017). How introns enhance gene expression. Int J Biochem Cell Biol.

[CR102] Shibata N, Kakeshita H, Igarashi K, Takimura Y, Shida Y, Ogasawara W, Koda T, Hasunuma T, Kondo A (2021). Disruption of alpha-tubulin releases carbon catabolite repression and enhances enzyme production in *Trichoderma reesei* even in the presence of glucose. Biotechnol Biofuels.

[CR103] Singh A, Taylor LE, Vander Wall TA, Linger J, Himmel ME, Podkaminer K, Adney WS, Decker SR (2015). Heterologous protein expression in *Hypocrea jecorina*: a historical perspective and new developments. Biotechnol Adv.

[CR104] Sinitsyn AP, Rozhkova AM, Kamm B (2015). *Penicillium canescens* host as the platform for development of a new recombinant strain producers of carbohydrases. Microorganisms in biorefineries.

[CR105] Sinitsyn AP, Volkov PV, Rubtsova EA, Shashakov IA, Rozhkova AM, Sinitsyna OA, Kondrat’eva EG, Zorov IN, Satrudinov AD, Merzlov DA, Matys VY (2018). Using an inducible promoter of the glucoamylase gene to construct new multienzyme complexes from *Penicillium verruculosum*. Catal Ind.

[CR106] Sonderegger C, Galgóczy L, Garrigues S, Fizil Á, Borics A, Manzanares P, Hegedüs N, Huber A, Marcos JF, Batta G, Marx F (2016). A *Penicillium chrysogenum*-based expression system for the production of small, cysteine-rich antifungal proteins for structural and functional analyses. Microb Cell Fact.

[CR107] Spiro RG (2002). Protein glycosylation: nature, distribution, enzymatic formation, and disease implications of glycopeptide bonds. Glycobiology.

[CR108] Stock J, Sarkari P, Kreibich S, Brefort T, Feldbrügge M, Schipper K (2012). Applying unconventional secretion of the endochitinase Cts1 to export heterologous proteins in *Ustilago maydis*. J Biotechnol.

[CR109] Sun X, Su X (2019). Harnessing the knowledge of protein secretion for enhanced protein production in filamentous fungi. World J Microbiol Biotechnol.

[CR110] Sun Y, Qian Y, Zhang J, Yao C, Wang Y, Liu H, Zhong Y (2022). Development of a novel expression platform for heterologous protein production via deleting the p53-like regulator Vib1 in *Trichoderma reesei*. Enzyme Microb Technol.

[CR111] Suzuki T, Dohra H, Omae S, Takeshima Y, Choi JH, Hirai H, Kawagishi H (2014). Heterologous expression of a lectin from *Pleurocybella porrigens* (PPL) in *Phanerochaete sordida* YK-624. J Microbiol Methods.

[CR112] Tanaka M, Tokuoka M, Shintani T, Gomi K (2012). Transcripts of a heterologous gene encoding mite allergen Der f 7 are stabilized by codon optimization in *Aspergillus oryzae*. Appl Microbiol Biotechnol.

[CR113] Tanaka M, Tokuoka M, Gomi K (2014). Effects of codon optimization on the mRNA levels of heterologous genes in filamentous fungi. Appl Microbiol Biotechnol.

[CR114] Te’o VSJ, Cziferszky AE, Bergquist PL, Nevalainen KMH (2000). Codon optimization of xylanase gene *xynB* from the thermophilic bacterium *Dictyoglomus thermophilum* for expression in the filamentous fungus *Trichoderma reesei*. FEMS Microbiol Lett.

[CR115] Terfrüchte M, Wewetzer S, Sarkari P, Stollewerk D, Franz-Wachtel M, Macek B, Schlepütz T, Feldbrügge M, Büchs J, Schipper K (2018). Tackling destructive proteolysis of unconventionally secreted heterologous proteins in *Ustilago maydis*. J Biotechnol.

[CR116] Toghueo RMK, Boyom FF (2020). Endophytic *Penicillium* species and their agricultural, biotechnological, and pharmaceutical applications. 3 Biotech.

[CR117] Tokuoka M, Tanaka M, Ono K, Takagi S, Shintani T, Gomi K (2008). Codon optimization increases steady-state mRNA levels in *Aspergillus oryzae* heterologous gene expression. Appl Environ Microbiol.

[CR118] Tomico-Cuenca I, Mach RL, Mach-Aigner AR, Derntl C (2021). An overview on current molecular tools for heterologous gene expression in *Trichoderma*. Fungal Biol Biotechnol.

[CR119] Uchima CA, Tokuda G, Watanabe H, Kitamoto K, Arioka M (2011). Heterologous expression and characterization of a glucose-stimulated β-glucosidase from the termite *Neotermes koshunensis* in *Aspergillus oryzae*. Appl Microbiol Biotechnol.

[CR120] Upshall A, Kumar AA, Bailey MC, Parker MD, Favreau MA, Lewison KP, Joseph ML, Maraganore JM, McKnight GL (1987). Secretion of active human tissue plasminogen activator from the filamentous fungus *Aspergillus nidulans*. Nat Biotechnol.

[CR121] van den Brink H(J)M, Petersen SG, Rahbek-Nielsen H, Hellmuth K, Harboe M (2006). Increased production of chymosin by glycosylation. J Biotechnol.

[CR122] Vavilova EA, Antonova SV, Barsukov ED, Vinetskii YP (2003). Mechanism of overproduction of secreted enzymes in the mycelial fungus *Penicillium canescens*. Appl Biochem Microbiol.

[CR123] Visser H, Joosten V, Punt PJ, Gusakov AV, Olson PT, Joosten R, Bartels J, Visser J, Sinitsyn AP, Emalfarb MA, Verdoes JC, Wery J (2011). Development of a mature fungal technology and production platform for industrial enzymes based on a *Myceliophthora thermophila* isolate, previously known as *Chrysosporium lucknowense* C1. Ind Biotechnol.

[CR124] Wakai S, Yoshie T, Asai-Nakashima N, Yamada R, Ogino C, Tsutsumi H, Hata Y, Kondo A (2014). L-lactic acid production from starch by simultaneous saccharification and fermentation in a genetically engineered *Aspergillus oryzae* pure culture. Bioresour Technol.

[CR125] Wang J, Mai G, Liu G, Yu S (2013). Molecular cloning and heterologous expression of an acid-stable endoxylanase gene from *Penicillium oxalicum* in *Trichoderma reesei*. J Microbiol Biotechnol.

[CR126] Wang J, Zeng D, Liu G, Wang S, Yu S (2014). Truncation of a mannanase from *Trichoderma harzianum* improves its enzymatic properties and expression efficiency in *Trichoderma reesei*. J Ind Microbiol Biotechnol.

[CR127] Wang Y, Li Z, Li Z, Bao X (2019). Over-expression of *Bgl1* from *Aspergillus*
*niger* in *Penicillium oxalicum*. AIP Conf Proc.

[CR128] Wang Q, Zhong C, Xiao H (2020). Genetic engineering of filamentous fungi for efficient protein expression and secretion. Front Bioeng Biotechnol.

[CR129] Ward PP, Lo JY, Duke M, May GS, Headon DR, Conneely OM (1992). Production of biologically active recombinant human lactoferrin in *Aspergillus oryzae*. Nat Biotechnol.

[CR130] Wei W, Chen L, Zou G, Wang Q, Yan X, Zhang J, Wang C, Zhou Z (2013). N-glycosylation affects the proper folding, enzymatic characteristics and production of a fungal β-glucosidase. Biotechnol Bioeng.

[CR131] Wiebe MG, Karandikar A, Robson GD, Trinci APJ, Candia JLF, Trappe S, Wallis G, Rinas U, Derkx PMF, Madrid SM, Sisniega H, Faus I, Montijn R, van den Hondel CAMJJ, Punt PJ (2001). Production of tissue plasminogen activator (t-PA) in *Aspergillus niger*. Biotechnol Bioeng.

[CR132] Wohlschlager L, Csarman F, Zrilić M, Seiboth B, Ludwig R (2021). Comparative characterization of glyoxal oxidase from *Phanerochaete chrysosporium* expressed at high levels in *Pichia pastoris* and *Trichoderma reesei*. Enzyme Microb Technol.

[CR133] Wu Y, Sun X, Xue X, Luo H, Yao B, Xie X, Su X (2017). Overexpressing key component genes of the secretion pathway for enhanced secretion of an *Aspergillus niger* glucose oxidase in *Trichoderma reesei*. Enzyme Microb Technol.

[CR134] Xu J, Gong ZZ (2003). Intron requirement for AFP gene expression in *Trichoderma viride*. Microbiology (n y).

[CR135] Xu Y, Wang Y-h, Liu T-q, Zhang H, Zhang H, Li J (2018). The GlaA signal peptide substantially increases the expression and secretion of α-galactosidase in *Aspergillus niger*. Biotechnol Lett.

[CR136] Yang L, Lübeck M, Lübeck PS (2017). *Aspergillus* as a versatile cell factory for organic acid production. Fungal Biol Rev.

[CR137] Yoon J, Aishan T, Maruyama JI, Kitamoto K (2010). Enhanced production and secretion of heterologous proteins by the filamentous fungus *Aspergillus oryzae* via disruption of vacuolar protein sorting receptor gene *AoVps10*. Appl Environ Microbiol.

[CR138] Yoon J, Maruyama JI, Kitamoto K (2011). Disruption of ten protease genes in the filamentous fungus *Aspergillus oryzae* highly improves production of heterologous proteins. Appl Microbiol Biotechnol.

[CR139] Yoon J, Kikuma T, Maruyama JI, Kitamoto K (2013). Enhanced production of bovine chymosin by autophagy deficiency in the filamentous fungus *Aspergillus oryzae*. PLoS One.

[CR140] Yu CH, Dang Y, Zhou Z, Wu C, Zhao F, Sachs MS, Liu Y (2015). Codon usage influences the local rate of translation elongation to regulate co-translational protein folding. Mol Cell.

[CR141] Zubieta MP, Contesini FJ, Rubio MV, de Souza Schmidt Gonçalves AE, Gerhardt JA, Prade RA, de Lima Damasio AR (2018). Protein profile in *Aspergillus nidulans* recombinant strains overproducing heterologous enzymes. Microb Biotechnol.

